# The impacts of rent burden and eviction on mortality in the United States, 2000–2019

**DOI:** 10.1016/j.socscimed.2023.116398

**Published:** 2024-01

**Authors:** Nick Graetz, Carl Gershenson, Sonya R. Porter, Danielle H. Sandler, Emily Lemmerman, Matthew Desmond

**Affiliations:** a Department of Sociology, Princeton University, Princeton, NJ, USA; b Center for Economics Studies, United States Census Bureau, Washington D.C., USA

## Abstract

Investments in stable, affordable housing may be an important tool for improving population health, especially in the context of rising costs and evictions for American renters. Still, a lack of longitudinal data linking these exposures to health outcomes has limited prior research. In this study, we use linked administrative data to estimate the associations of rent burden and eviction with all-cause mortality. We constructed a novel dataset linking renters in the long-form 2000 Census (N = 6,587,000) to mortality follow-up through 2019 from the Census Numident file. To measure exposure to eviction, we further linked this dataset to 38 million eviction records between 2000 and 2016 using names and addresses. For a subsample of renters, we also measured within-individual changes in rent burden between 2000 and 2008–2012 by linking to the American Community Survey. We estimated the associations of rent burden and eviction with mortality using Cox proportional-hazards models and discrete-time hazard models adjusted for individual, household, neighborhood, and state characteristics, examining varying associations by cohort, race, gender, and eviction risk. Higher baseline rent burden, increases in rent burden during midlife, and evictions were all associated with increased mortality. Compared to a baseline rent burden of 30%, a burden of 70% was associated with 12% (95% confidence interval = 11–13%) higher mortality. A 20-point increase in rent burden between 2000 and 2008–2012 was associated with 16% (12–19%) higher mortality through 2019. An eviction filing without judgment was associated with a 19% (15–23%) increase in mortality and an eviction judgment was associated with a 40% (36–43%) increase. Associations were larger for those at lower predicted risk of eviction. These findings reveal how rising costs and evictions are shaping mortality for American renters. Policies designed to increase the supply of affordable housing and prevent eviction may lead to widespread improvements in population health.

## Highlights

• We describe a novel linked dataset including eviction and mortality records.
• Increases in rental costs are associated with increases in mortality risk.
• Eviction filings and judgments are both associated with increased mortality.
• Impacts of eviction on mortality are largest for those at lower eviction risk.

The study of housing as a social determinant of health has a long history (Leifheit et al., 2022; Engels, 2009; Du Bois, 1899; Hernández et al., 2019) with much of this research focusing on the quality and safety of housing (e.g., lead paint) and neighborhood effects (e.g., segregation) (Williams and Collins, 2001; Jacobs, 2011). However, there has been less attention paid to the economic and legal aspects of housing and their relationship to health, especially for renters (Sewell, 2016, 2021; Reynolds, 2021). Despite stagnant wages, rental prices have more than doubled over the last two decades (Myers and Park, 2019). In 2020, half of poor renter households were severely rent burdened, with over 50% of income dedicated to rent—only to see rents increase at the fastest rate ever recorded in the following year (Bhattarai et al., 2022). Each year, 2.7 million households—roughly 7% of all renter households—face the threat of eviction (Gromis et al., 2022).

Homeowners benefit from predictable, fixed-rate mortgage payments, while renters face substantial volatility in housing costs due to the ability of most American landlords to raise rents at their discretion. In some markets, rent hikes in recent years have been dramatic, with average rents increasing by 26% in Gaston County, North Carolina, 28% in Maricopa County, Arizona, and 39% in Collier County, Florida, between 2019 and 2022 (Bhattarai et al., 2022). As rents rise, health-related spending is often crowded out. Poor households with children who are moderately rent-burdened (i.e., 30–50% of income dedicated to rent) spend 57% less on healthcare and 17% less on food compared to unburdened households (Airgood-Obrycki et al., 2022).

Households prioritize rent over other essential spending in part because landlords have the power to evict tenants. Eviction can be a disruptive event that leads to disenrollment from social safety net programs such as Medicaid, job loss, and a host of other negative consequences (Schwartz et al., 2022; Desmond and Kimbro, 2015; Collinson et al., 2022). Eviction can also compromise a person's physical and mental health by exposing them to prolonged periods of intense housing precarity, including homelessness, and acute stress (Desmond and Kimbro, 2015). In addition, eviction can increase exposure to infectious disease, as seen during the COVID-19 pandemic (Benfer et al., 2021; Leifheit et al., 2021; Sandoval-Olascoaga et al., 2021). The consequences of eviction—exposure to residential segregation, concentrated poverty, and labor precarity among them—have been established as “fundamental causes” of population health, influencing the distribution and life course trajectories of chronic disease, adverse health events, and death (Williams and Collins, 2001; Phelan and Link, 2015; Link and Phelan, 1995).

In this study, our objective was to estimate how rent burden (the percentage of a household income that is spent on rent) and eviction events (eviction filing without judgment, eviction judgment) are related to all-cause mortality in the United States from 2000 to 2019, adjusting for an extensive set of individual, household, neighborhood, and state characteristics. We constructed a novel longitudinal dataset linking individual renters from the long-form 2000 Census to 38 million eviction court records from 2000 to 2016, the American Community Survey (ACS) from 2008 to 2012, and administrative death records from the Census Numident file from 2000 to 2019. We analyzed the associations of rent burden and eviction events with mortality risk using Cox proportional-hazards models and discrete-time hazard models, examining how these associations varied by age, race, ethnicity, gender, and estimated eviction risk. Overall, this study provides evidence that housing cost burden and evictions are an important dimension for understanding mortality among American renters.

## 1 Background

### 1.1 The political economy of housing as a fundamental cause of health

We consider the political economy of housing, particularly the economic and legal relations associated with renting (e.g., rental costs and evictions), within the fundamental cause theory of population health. The deployment of flexible resources—money, knowledge, power, prestige, social networks—is central to fundamental cause theory, as the specific mechanisms through which these resources are used in relation to health can be easily substituted over time and space (Link and Phelan, 1995). Those with flexible resources can more easily adapt to new settings of disease burden; for example, more easily accessing new health-enhancing technologies and behaviors (e.g., vaccines, working from home) for an emergent disease risk (e.g., COVID-19).

There has been some emphasis on housing within the fundamental cause framework, such that housing inequality puts certain individuals at higher “risk of risks”—for example, residential segregation patterns exposure to low-quality housing stock, physical hazards, and proximity to healthcare services (Williams et al., 2019). But in frameworks that treat socioeconomic position as a fundamental cause of health outcomes, characteristics such as “renter” or “owner” are largely treated as “upstream” features of the individual, in the sense that individuals derive different flexible resources from these statuses (money, social networks) and translate these to different health-relevant resources (healthcare, nutrition).

There has been less focus on the relational systems that maintain these connections and give these socioeconomic positions meaning, (Reynolds, 2021; Emirbayer, 1997; Muntaner, 2013; Williams, 2019) which is particularly important for considering the influence of renting conditions on health. Reynolds (2021) draws insights from the political economy of health tradition to describe a framework of *health power resources*. This framework focuses on how broader forms of power consolidation and power relations between specific actors organize both the distribution of socioeconomic and flexible resources via stratification (e.g., who is able to access favorable mortgage terms and high value homeownership vs. who is left to seek housing in racialized rental submarkets) and the translation of those resources to health via the commodification of health-relevant resources (e.g., largely privatized healthcare), material deprivation resulting from discrimination (e.g., segregation of rental costs and eviction risk in rental submarkets), and reduced efficacy resulting from devitalization (e.g., propensity to engage in risky health behaviors). In Fig. S1, we map these concepts onto the power relations underpinning housing stratification and mortality. Power consolidation in the housing market and policy stratifies ownership and translates the status of renting to increased mortality risk through many different mechanisms. In this paper, we focus on two important economic and legal mechanisms: rent burden and eviction.

Research broadly focused on the unequal effects of disruptive events (e.g., loss of employment, divorce) suggests that the consequences of eviction for mortality might vary across dimensions such as race, gender, and the likelihood of receiving an eviction judgment (Aquino et al., 2022). In this study, we test two diverging explanations as to why we might find varying impacts of eviction given the fact that the distribution of renting conditions and eviction is very racially stratified in the United States (Hepburn et al., 2020; Krysan and Crowder, 2017). Black renters live in worse renting conditions on average than white renters, contributing to differential baseline mortality between these populations (Desmond and Wilmers, 2019; Massey and Denton, 1993). On one hand, this implies we might find larger impacts of eviction on mortality for those at high risk of eviction (e.g., Black renters) due to existing resource disparities and cumulative disadvantage that make it more difficult to navigate the event of an eviction. On the other hand, we might expect to find larger impacts for those at relatively low risk of eviction (“negative selection”) where an eviction may be a more unexpected and non-normative shock to baseline mortality rates that are relatively lower, such as among white renters (Aquino et al., 2022).

### 1.2 Research objectives

We have two objectives in our analysis of how the political economy of housing shapes mortality risk for American renters. First, we hypothesize the increased rents and eviction events are associated with increased mortality. Second, we hypothesize that the association between eviction events and mortality varies by cohort, race, gender, and eviction risk. Testing these hypotheses requires constructing new linked data, especially because administrative data from eviction court systems contain very limited information about each eviction case, typically only the names and address of defendants. Data linkage is therefore necessary to observe characteristics of those filed against and track outcomes.

## 2 Data and methods

### 2.1 Linking decennial census data to administrative death records

To construct our baseline sample, we used the long-form 2000 Decennial Census, which was completed by roughly 16% of the U.S. population. The Census allowed us to measure a set of baseline individual, household, neighborhood, and state characteristics that may confound the relation between our primary exposure measures—level of rent burden in 2000, change in rent burden from 2000 to 2008–2012, eviction filing without judgment, eviction filing with judgment—and our primary outcome, all-cause mortality.

To measure all-cause mortality, we obtained individual death records from the Census Numident file, which contains all interactions related to social security numbers (SSNs) that individuals have had with the Social Security Administration since 1972. This includes information on applications for SSNs, requested changes to SSN information, and date of death. The Numident file is the system of record for death information within the Social Security Administration. It also provides the foundation for the Personal Identification Validation System, which the Census Bureau uses to assign a unique identifier called a Protected Identification Key (PIK) to all individuals based on social security number, name, date of birth, address, and sex as available (Finlay and Genadek, 2021; Wagner and Layne, 2014). We used the Census Numident all-cause mortality data for this research, as opposed to cause-specific data from the Centers for Disease Control and Prevention, because it has been shown to be of high quality and is also a part of the Census Bureau's data linkage infrastructure, which allows us to link death information from this file to other administrative records and survey data. PIKs enable us to link records at the person-level over time and across Census survey and administrative sources—as well as to any other external data source that can be reliably assigned PIKs. We merged dates of death from the Numident file to virtually all renters in the 2000 Census using PIKs, allowing us to measure all-cause mortality from 2000 to 2019.

### 2.2 Exposure to baseline rent burden and rent burden changes in midlife

We measured our first exposure—baseline rent burden—among all renters in the 2000 Census. To measure our second exposure—within-individual rent burden change—we created a subsample by merging renters from the 2000 Census to the 2008–2012 American Community Survey (ACS) by PIK. The ACS is only distributed to roughly 3.5 million households each year, so we pooled ACS data across these five adjacent years to avoid issues arising from small sample sizes. We used tenure status observed in the ACS to identify individuals still renting roughly a decade after completing the 2000 Census.

To analyze the association between levels of rent burden and mortality, we focused on the 2.1 million renters who were middle-aged (ages 40–65) in 2000. Similarly, we focus on the 93,000 persistent renters who were middle-aged in 2008–2012 for exposure to rent burden changes since 2000 (i.e., rent burden changes experienced from ages 30–55 to 40–65). This allowed us to analyze these associations during a period of the life course prior to retirement ages (when there is higher risk of mortality selection) and after young working ages (when income and tenure are more volatile).

### 2.3 Exposure to eviction filing and judgment

To estimate the association between eviction events and mortality, we used a national database of eviction records compiled by the Eviction Lab at Princeton University (Desmond et al., 2018a, b) (Fig. S2, Fig. S3). We submitted eviction records from 2000 to 2016 (58 million records) to the Census Bureau's Personal Identification Validation System, which assigned PIKs to individual defendants using a probabilistic linkage based on first name, last name, and address reported in eviction filings; doing so resulted in 38 million matches based on a 65% match rate (Census Disclosure Review Board Approval Number: CBDRB-FY23-CES004-013; sample sizes are rounded according to Census disclosure policy). We merged matched defendants to the 2000 Census by PIK and subset to those renting and age 22 or older at baseline (6.6 million renters, including 187,000 renters who received at least one eviction filing but never a judgment and 327,000 renters who received an eviction judgment between 2000 and 2016). The Supplementary Information includes more details on the construction of all samples (Fig. S4, Section S1).

### 2.4 Analytic strategy

To assess the association between baseline rent burden in 2000 and mortality from 2000 to 2019, we estimated hazard ratios using Cox proportional-hazards models adjusting for a set of individual, household, neighborhood, and state characteristics measured in 2000. These potential confounding factors included baseline age, race, ethnicity, gender, educational attainment, household income, nativity, number of children, household size, marital status, living in the same place five years ago, veteran status, disability status, being unemployed, number of bedrooms, residential building size, tract-level median household income, tract-level poverty rate, and state of residence. We used similar Cox models to assess the association between within-individual changes in rent burden (2000–2008–2012) and mortality (2008–2012 to 2019). For these models, we also adjusted for income trajectories from 2000 to 2008–2012 to isolate mortality risk associated with rent burden fluctuations due to changes in *rent* rather than changes in *income* (e.g., changes in wages or household formation). In sensitivity analyses, we estimated associations based on subsamples where we removed individuals who moved over the period and individuals who experienced large decreases in household income over the period.

To assess the associations between time-varying eviction events (filing without judgment, filing with judgment) and mortality between 2000 and 2016, we estimated hazard ratios using discrete-time hazard models with the complementary log-log link adjusted for the same set of confounders above. We also estimated hazard ratios based on models stratified by cohort, race-ethnicity-gender, and eviction propensity. We used eviction propensity, which combined information across all our covariates on the likelihood of experiencing an eviction between 2000 and 2016, to examine possible “negative selection” into eviction. Negative selection refers to the fact that the population targeted for eviction was likely already disadvantaged in many other ways and may have some of the highest background mortality rates (Aquino et al., 2022; Brand and Xie, 2010). As such, the estimated hazard ratios between eviction events and mortality may be relatively lower for tenants at the highest risk of eviction exposure, owing to a combination of ceiling effects and the fact that eviction may be a more unexpected and non-normative shock for the population at lowest risk of exposure (i.e., where background mortality rates are relatively lower). (Aquino et al., 2022).

As a result of internal imputation procedures conducted by the Census Bureau there are no missing covariate data (U.S. Census Bureau, 2003). The proportional-hazard assumptions were tested and verified by the inclusion of an interaction term with time in the model. See the Supplementary Information for more details on the specification of mortality models (Section 1, 2) and limitations (Section S2.4).

## 3 Results

### 3.1 Descriptive statistics

Characteristics of renters in the 2000 Census (n = 6,587,000) are presented by eviction status in Table 1. Between 2000 and 2016, we observed 180,000 of these renters receive an eviction filing that did not result in a judgment and 327,000 receive an eviction judgment. We recorded 1,474,400 deaths among renters never filed against, 27,000 deaths among renters filed against without a judgment, and 45,000 deaths among renters evicted.

**Table 1:** **Characteristics of the study population at baseline in 2000, stratified by eviction filing status over the period 2000**–**2016.** Sample sizes and death counts are rounded according to Census disclosure policy. Census Disclosure Review Board Approval Number: CBDRB-FY23-CES004-016.

	Never filed against	Filed against without a judgment	Evicted (filed against with a judgment)
Individuals	6,085,518	180,000	327,000
Number of events (deaths)	1,474,400	27,000	45,000
Race-ethnicity-gender, %			
Non-Hispanic white men	31.1	16.9	20.2
Non-Hispanic white women	35.2	18.7	23.5
Non-Hispanic Black men	5.3	13.8	12.0
Non-Hispanic Black women	7.5	29.6	25.6
Hispanic men	3.2	2.7	2.5
Hispanic women	3.4	4.1	3.3
Asian	4·5	1.8	1.3
American Indian and Alaskan Native	1.2	0.9	1.4
Native Hawaiian or Pacific Islander	0.2	0.1	0.2
Other race/ethnicity	8.5	11.4	10.1
Educational attainment, %			
College or more	26.6	17.3	12.3
Some college	22.4	25.5	26.1
High school	28.1	31.5	34.8
Less than high school	22.9	25.8	26.8
Size of rental property, %			
1 unit, mobile	5.4	3.4	5.1
1 unit, single-family	39.1	27.0	35.5
2 units	9.4	8.7	9.4
3–4 units	10.8	10.8	12.2
5–9 units	10.2	12.2	12.2
10–19 units	8.4	10.8	9.4
20–49 units	6.7	10.9	6.9
50+ units	10.1	16.2	9.4
Household income, median	32,200	29,000	27,000
Monthly rent as % of income, mean (SD)	26.6 (23.6)	31.1 (25.9)	32.1 (26.1)
Same home for last five years, %	32.9	32.7	23.0
Number of bedrooms, mean (SD)	2.1 (1.0)	2.0 (1.0)	2.1 (1.0)
Number of children, mean (SD)	0.7 (1.1)	1.0 (1.3)	1.2 (1.3)
Household size, mean (SD)	2.1 (2.0)	2.6 (2.0)	2.8 (2.0)
U.S.-born, %	82.7	81.6	88.5
Disability, %	26.9	28.0	26.6
Veteran, %	11.2	8.3	9.2
Unemployed, %	4.2	7.7	9.0
Married, %	42.1	33.0	34.6
Tract household income, median	46,110	40,810	40,200
Tract poverty rate, median	15.9	20.9	19.7

Many Americans who were persistently renting during midlife (2000 to 2008–2012) experienced decreases in rent burdens (Fig. S5). Still, a substantial proportion of both poor and non-poor renters experienced increases in their rent burden over the same period. This was especially true for poor renters, where 47% experienced an increase in rent burden of 5 percentage points or more by 2008–2012. For many poor renters, these increases were much larger. For example, among poor renters with a rent burden of 30–49% in 2000, 45% experienced an increase of 20 percentage points or more by 2008–2012 (Fig. S5).

### 3.2 Baseline levels of rent burden and mortality risk

**Fig. 1:**
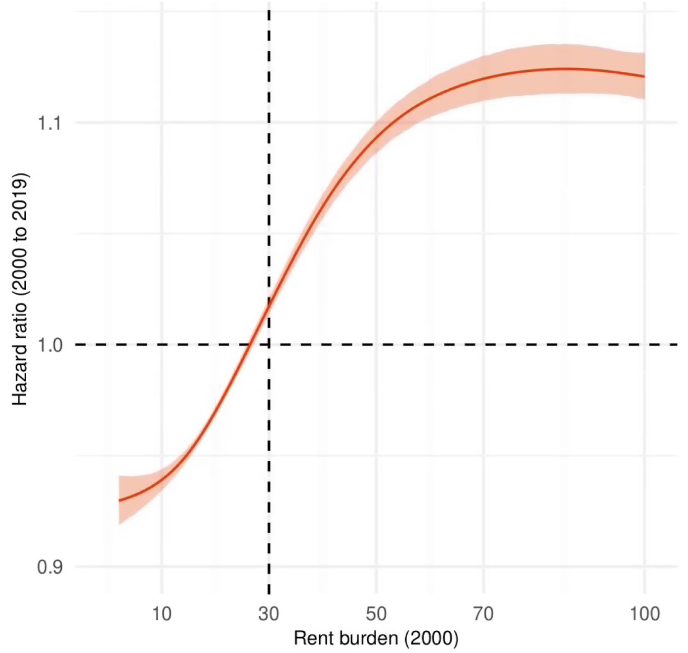
**Adjusted hazard ratio for all-cause mortality from 2000**–**2019 associated with changes in baseline rent burden in 2000.** Estimates of the hazard ratio are reported based on a penalized cubic spline for continuous rent burden centered on 30% (the dashed lines at rent burden of 30% and a hazard ratio of 1.0 indicates the reference); Fig. S3 includes estimates of the hazard ratio based on a linear term. Census Disclosure Review Board Approval Number: CBDRB-FY23-CES004-016. Sources: 2000 Census linked to 2021 Numident file.

Higher rent burdens in 2000 were associated with increased mortality through 2019, with a steep gradient in the adjusted hazard ratio between 20% and 70% rent burden (Fig. 1 reports estimates based on a penalized cubic spline for continuous rent burden; Table S1 and Fig. S6 include estimates based on a linear term). Compared to a rent burden of 30%, a rent burden of 50% was associated with 9% higher mortality from 2000 to 2019 (hazard ratio [HR] = 1.09, 95% confidence interval [CI] = 1.09, 1.10) (p < 0.001), and a rent burden of 70% was associated with 12% higher mortality (HR = 1.12, CI = 1.11, 1.13) (p < 0.001). Analyses stratified by race-ethnicity-gender indicated that the association between high rent burdens and increased mortality was pronounced for men, especially for Hispanic men and non-Hispanic Black men, where a rent burden of 70% compared to 30% was associated with a mortality increase of 16% (HR = 1.16, CI = 1.11, 1.22) (p < 0.001) and 14% (HR = 1.14, CI = 1.11, 1.17) (p < 0.001), respectively (Fig. S7).

### 3.3 Changes in rent burden and mortality risk

**Fig. 2:**
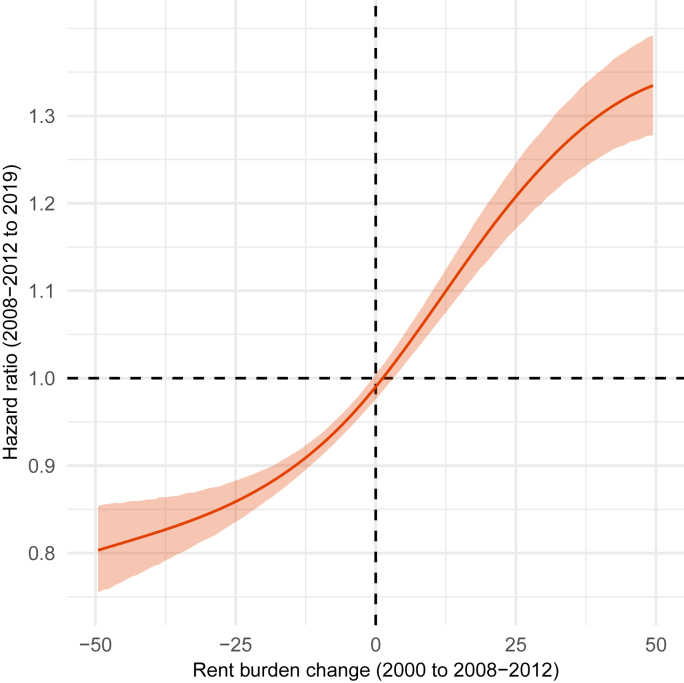
**Adjusted hazard ratio for all-cause mortality from 2008**–**2012 to 2019 associated with changes in rent burden from 2000 to 2008**–**2012.** Estimates of the hazard ratio are reported based on a penalized cubic spline for continuous rent burden change centered on 0.0 (the dashed lines at rent burden change of 0.0 and hazard ratio of 1.0 indicate the reference); Fig. S5 includes estimates of the hazard ratio based on a linear term. Census Disclosure Review Board Approval Number: CBDRB-FY24-CES004-001. Sources: 2000 Census and 2008–2012 ACS linked to 2021 Numident file.

For middle-aged renters in the 2008–2012 ACS who had been renting a decade prior in the 2000 Census, a 10-point increase in rent burden over that period was associated with 8% higher mortality (HR = 1.08, CI = 1.05, 1.10) (p < 0.001) compared to no increase in rent burden while a 20-point increase was associated with 16% higher mortality (HR = 1.16, CI = 1.12, 1.19) (p < 0.001) (Fig. 2, Table S1). In sensitivity analyses, we estimated associations based on the subsample of renters that we observed living in the same Census block in both 2000 and 2008–2012 (33,000 renters) to exclude rent increases due to moves. We use Census block to determine non-movers, which means we may be including some individuals who did move within blocks over the period, but we suspect such moves are rare. We find that estimated hazard ratios are similar for this subsample (Fig. S8).

### 3.4 Eviction events and mortality risk

**Fig. 3:**
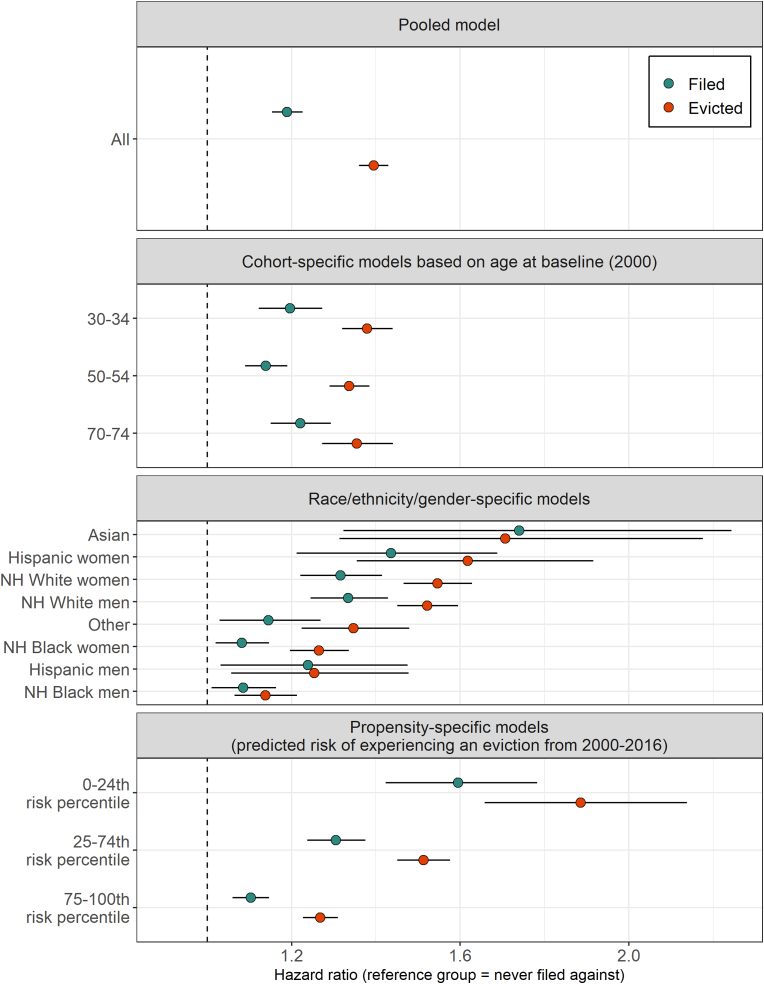
**Adjusted hazard ratios for all-cause mortality from 2000**–**2016 associated with eviction events.** Eviction events include an eviction filing that did not result in a judgment and an eviction judgment. The dashed line at a hazard ratio of 1.0 indicates the reference group of renters never filed against. Census Disclosure Review Board Approval Number: CBDRB-FY23-CES004-013. Sources: 2000 Census linked to 2021 Numident file and 2000–2016 eviction records.

Across all renters, the threat of eviction—a court filing without judgment—was associated with a 19% increase in mortality (HR = 1.19, CI = 1.15, 1.23) (p < 0.001) and an eviction—receiving a judgment—was associated with a 40% increase (HR = 1.40, CI = 1.36, 1.43) (p < 0.001) (Fig. 3, Table S1). In analyses stratified by cohort, we find that despite much higher background mortality rates at older ages, hazard ratios are similar across ages 30–34, 50–54, and 70–74 in 2000. In analyses stratified by race-ethnicity-gender, eviction was associated with higher mortality across all groups and associations tended to be larger for groups where eviction was less common (e.g., white renters).

Analyses stratified by estimated eviction risk indicated negative selection into eviction (Fig. 3, Table S1): the association between eviction and mortality was significantly larger for those estimated to be in the lowest eviction risk quartile (HR = 1.89, CI = 1.66, 2.14) (p < 0.001). Still, we found that an eviction judgment was associated with a 27% increase in mortality even among those estimated to be at highest risk (HR = 1.27, CI = 1.23, 1.31). See Section S3 for additional sensitivity analyses.

## 4 Discussion

In a study matching millions of housing court records to decennial census, survey, and administrative data, we found that rent burden and eviction events were significantly associated with higher mortality risk. Notably, we found not only that rent burdens in excess of 30%—the common threshold of “rent-burdened”—are associated with increased mortality, but also that rent burdens below 30% are similarly protective. By using longitudinal data to observe within-individual rent burden increases over a decade, we found that the association between rent burden and mortality was significant even when analyzing only those who rented in the same place over time, suggesting that the association was not driven by selection into higher rents via moves. These rent increases may be considered a plausibly exogenous shock to renters since they were unlikely to depend on the characteristics or behaviors of the renters themselves. Rather, it is likely a large proportion of renters obtain housing in submarkets where rents continue to increase throughout midlife, far outpacing wage growth (Desmond, 2018). This may force renters to prioritize housing costs over other health-related spending such as preventative healthcare (Airgood-Obrycki et al., 2022; Desmond and Kimbro, 2015; Benfer et al., 2021).

A report from the Department of Housing and Urban Development in 2012 investigated households with severe rent burdens and described a “puzzling” relationship between rent burden and household mobility. The report found that households with severe rent burdens were no more likely to move in the following years than those with lower burdens (Eggers and Moumen, 2012). However, this lack of responsive moves may not be so puzzling after all. Due to substantial compression of rental prices (Desmond and Wilmers, 2019; Boeing and Waddell, 2016) and housing policies that have not kept pace with growing need, the supply of affordable housing has declined (National Low Income Housing Coalition, 2021). Consequently, low-income tenants are forced to adapt in place to rent hikes when resources are constrained, which may ultimately increase their mortality risk.

Eviction filings and judgments were associated with 19% and 40% increases in mortality risk, respectively. Most evictions occurred within highly marginalized renter populations where mortality rates were already very high. However, even when we estimated associations within the most disadvantaged renters—those estimated to be at highest risk of experiencing an eviction—we found that eviction was still associated with a 27% increase in mortality. The well-documented negative consequences of eviction, such as homelessness and “downward moves” that involve families relocating to neighborhoods with higher crime and poverty rates, can have an impact on one's health. Future research and data linkage will be needed to document which factors (e.g., homelessness, healthcare access, etc.) may be the most influential mechanisms connecting eviction to increased mortality risk.

This study found high rent burdens and evictions are hazardous to health. Therefore, public policies designed to increase the supply of affordable housing—such as rental vouchers and small-dollar mortgages—and to prevent eviction—such as diversion programs and legal aid—may lead to improvements for public health. These policies may also help reduce racial disparities in health and mortality, given that Black and Hispanic families disproportionately rent their homes due to the nation's legacy of redlining and mortgage discrimination (Taylor, 2019; Rothstein, 2017).

## Code availability

The final code used to conduct our analyses is available here: https://github.com/ngraetz/ssm_rent_eviction_mortality/.

## Declaration of competing interest

The authors declare no competing interests.

## Data Availability

Researchers may apply for access to our confidential linked data from any of the 16 principal federal statistical agencies and units: https://www.census.gov/about/adrm/fsrdc.html.
