# Supplementary material for: The impacts of rent burden and eviction on mortality in the United States, 2000–2019
Source: Soc Sci Med. 2024 Jan;340:116398. doi: 10.1016/j.socscimed.2023.116398 (PMC10828546; doi:10.1016/j.socscimed.2023.116398)
Supplement: Multimedia component 1 [file mmc1.docx]

Supplementary Material

Table of Contents

- Section S1. Data sources and sample creation
- Section S2. Mortality models
- References for supplementary text
- Table S1. Associations of rent burden and eviction with mortality risk
- Figure S1. County-years with complete coverage of eviction court filings from 2000-2016
- Figure S2. Geographic variation in the average annual eviction filing rate, 2000-2016
- Figure S3. Theoretical model
- Figure S4. Sample creation
- Figure S5. Levels of rent burden in 2000 and changes in rent burden from 2000 to 2008-2012 for poor and non-poor persistent renters
- Figure S6. Comparison of functional forms for levels of rent burden
- Figure S7. Variation in the association between rent burden and mortality by race/ethnicity and gender
- Figure S8. Comparison of functional forms for rent burden change
- Figure S9. Sensitivity analysis for varying rates of eviction data coverage and PIK assignment

**S1. Data sources and sample creation**

*S1.1. Baseline data: 2000 Decennial Census records*

Our analyses require observing cohorts over time. We use the long-form 2000 Decennial Census (covering approximately 16% of the population) as our baseline, where we observe tenure (owning vs. renting) and our key demographic dimensions: race-ethnicity (non-Hispanic white, non-Hispanic Black, Hispanic, Asian, American Indian and Alaska Native, Native Hawaiian and Pacific Islander, and other), gender (men and women), and baseline age. We use the variables for race-ethnicity and gender included in the Census instrument to proxy exposure to contemporary and historical systems of gendered racialization and racism as they relate to rental costs, eviction, and premature mortality, the nuance of which are likely not captured by these limited survey categories. We also use the terms for different self-identified categories as they are reported in the Census (e.g., “Hispanic”).

We also observe an extensive set of potential confounders related to our exposures (rent burden, eviction) and outcome (mortality): educational attainment (less than high school, high school, some college, college or more), household income (continuous), rent burden (continuous), U.S.-born (0/1), number of children (continuous), household size (continuous), marital status (0/1), living in the same place five years ago (0/1), veteran status (0/1), disability status (0/1), unemployed (0/1), number of bedrooms (continuous), residential building size (single-unit, mobile single-unit, 2, 3-4, 5-9, 10-19, 20-49, 50 or more units), tract-level median household income (continuous), and tract-level poverty rate (continuous).

*S1.2. Outcome data: 2000-2019 Census Numident death records*

We draw individual mortality information from the Census Numident file, which contains all interactions related to social security numbers (SSNs) that individuals have had with the Social Security Administration (SSA) since 1972, including information on applications for SSNs, requested changes to SSN information, and death information. The SSA collects death information for the purposes of administering the Old-Age, Survivors, and Disability Insurance program, often referred to as “Social Security.” This death information is obtained from many different sources, including first-party reports of death from family members and representatives as well as verified third-party reports from friends, state government offices, the Centers for Medicare and Medicaid Services, the Department of Veterans Affairs, and the Internal Revenue Service. SSA undertook the Death Data Improvement Initiative in 2019 following a quality assessment of this death information, which resulted in cumulative updates and consolidation of death information in the Numident file going back to 1960. The Numident file is now the single system of record for death information in the SSA.(Finlay and Genadek, 2021)

Finlay and Genadek (2021) compared all-cause mortality from the Numident file to estimates derived from data collected by the Centers for Disease Control and Prevention (CDC), which are based on death certificates from state vital statistics offices that have been provided to the National Center for Health Statistics (NCHS). Importantly, the CDC estimates only include deaths occurring in U.S. states and are not limited to SSN holders, whereas the Census Numident includes deaths of SSN holders dying abroad and in U.S. territories but does not include deaths occurring in the United States for those without an SSN. Finlay and Genadek (2021) compare weekly death estimates across age of death and state, concluding that the timing and quality of the Census Numident data have improved over time and are a high-quality source for measuring all-cause mortality.

The Census Bureau, through the Personal Identification Validation System (PVS), assigns a unique, anonymous internal identifier called a Protected Identification Key (PIK) to all individuals based on social security number, name, date of birth, address, and sex as available.(Wagner and Layne, 2014) PIKs are used to link records at the person-level over time and across survey and administrative records in order to improve Census Bureau survey and decennial census data, to develop innovative data products, and conduct research. The ability to link the Census Numident data to other administrative records data, as well as the decennial censuses, American Community Survey (ACS), and other federal surveys, opens many avenues for understanding the relationships between mortality and demographic characteristics, social factors, economics, and geographic settings—as well as the linkage of all-cause mortality data to any other external data source that can be reliably assigned a PIK through PVS. It is important to note that there is bias in who receives a PIK. For instance, minorities, people with lower socioeconomic status, people who are not employed, immigrants, and recent movers are less likely to receive a PIK.(Bond et al., 2014)

We merge dates of birth and death from the Numident file to virtually all renters in the 2000 Census, allowing us to follow cohorts and observe all-cause mortality through 2019. We next merge data on our target exposures during the period, which requires constructing two samples: one for estimating associations with changes in rent burden and one for estimating associations with eviction (Figure S4).

*S1.3. Exposure to rent burden increases: 2008-2012 American Community Survey*

Our first rent exposure measured above in the 2000 Census is baseline levels of rent burden and the association with mortality from 2000-2019. Our second rent exposure is within-individual changes in rent burden over time. To estimate changes for a subsample of renters in 2000, we draw on rent burdens from the ACS, which is distributed to roughly 3.5 million households each year. We pool ACS data across five adjacent years (2008-2012) to avoid issues arising from small sample sizes. For those who report renting in the 2008-2012 ACS, we merge records to renters from the 2000 Census by PIK. To analyze the relationship between rent burden and mortality, we focus on those who were middle-aged (ages 40-65) in the year 2000 for analyzing the relationship between rent burden levels and mortality. Similarly, we focus on those who were middle-aged in the years 2008-2012 for rent burden changes (i.e., the rent burden changes were experienced from roughly ages 30-55 to 40-65). This allows us to observe a period prior to retirement ages (when there is higher risk of mortality selection) and after young working ages (when income and tenure are more volatile). This results in a subsample of renters in both the 2000 long-form Census and one year of the 2008-2012 ACS (N=93,000). For this analysis, we define the exposure as rent burden changes between 2000 and 2008-2012. The outcome is mortality following renters from the point of observation in the ACS (2008-2012) through 2019.

*S1.4. Exposure to eviction: 2000-2016 eviction court filings*

We draw on eviction records from 2000-2016 compiled by the Eviction Lab at Princeton University(Desmond et al., 2018a) (Figure S1, Figure S2). These records were collected, either manually or via bulk extracts from court administrative data systems, by LexisNexis Risk Solutions. They were cleaned, stripped of duplicate and commercial eviction cases, geocoded, and validated against publicly available data sources published by county and state court systems.(Desmond et al., 2018b) Court records provide an opportunity to examine the incidence of formal eviction across time and space. Studies based on court-ordered eviction records produce more accurate estimates than those reliant on self-reports in surveys.(Desmond, 2012) Although administrative data from court systems contain millions of records, they provide limited information about each case: case numbers, names of plaintiffs (e.g., landlords, property managers) and defendants (tenants), defendant addresses, and filing dates. Defendant gender and race/ethnicity are not recorded in eviction records. Administrative data linkage is therefore necessary to examine the varying impacts of eviction across subpopulations, as well as follow-up indicators in the years following eviction, such as mortality.

We submitted eviction records from 2000-2016 (58 million records) to the U.S. Census Bureau’s PVS, which assigned PIKs using a probabilistic linkage between records based on first name, last name, and address reported in eviction filings (38 million matches; 65% PIK match rate nationally; Census Disclosure Review Board Approval Number: CBDRB-FY23-CES004-013). We merge the eviction records to the 2000 Census by PIK (Census Disclosure Review Board Approval Number: CBDRB-FY23-CES004-013). We subset to those renting and age 22 or older at baseline in the 2000 Census (N=6.6 million renters, including N=187,000 renters who received at least one eviction filing but never a judgment and N=327,000 renters who received an eviction judgment from 2000-2016). We use this age threshold to avoid including the majority of college students. This threshold is arbitrary, but we did not find that setting the threshold at a slightly younger or older age substantively affected results. See Figure S4 for an illustration of sample creation.

There are two important features regarding these linked eviction data that may bias our estimates of the association between eviction events and mortality. First, the total database of 58 million records does not capture 100% of formal eviction cases from 2000-2016; coverage varies across both time and space. We define our microdata from a particular county-year as having complete coverage if we are able to validate the total count of filings in the microdata against aggregate filing statistics reported by county offices; see Gromis et al. (2022) and Desmond et al. (2018) for more information on this validation process. We have complete coverage for 39,493 county-years (72.1% of all possible county-years from 2000-2016); see Figure S2. This creates the possibility that some individuals included in our “never filed against” group (i.e., those we observe renting in the 2000 Census but never observe filed against from 2000-2016 in our linked records) were in fact filed against at some point in the 27.9% of county-years for which we do not observe all court filings. Second, we are only able to assign a PIK to roughly 65% of individuals included in the 58 million filings. Some of this may be due to clerical errors in the names and addresses listed on filings, but most is likely due to bias in who receives a PIK in the first place. For instance, minorities, people with lower socioeconomic status, people who are not employed, immigrants, and recent movers are less likely to receive a PIK.(Bond et al., 2014) This creates the possibility that we are excluding from our analysis many filings against tenants who are likely the most disadvantaged. We believe both these features likely result in a conservative bias for our estimates of the association between eviction events and mortality. Incomplete filing coverage causes us to miss some number of true eviction events and incorrectly treat those affected renters as never filed against. Incomplete PIK match rates cause us to exclude evictions among what is likely a very marginalized population altogether. See Limitations for further discussion (Section S3).

**S2. Mortality models**

*S2.1. Rent burden and all-cause mortality*

We use two exposures to describe the association of rent burden with all-cause mortality: *levels* of rent burden using the 2000 Census (N=2.1 million) and *changes* in rent burden using the subsample of all renters in the 2000 Census who were also sampled and renting in one year of the 2008-2012 ACS (N=93,000) (Figure S4).

First, we estimate the association between rent burden levels (2000) and all-cause mortality (2000-2019). For this model, we use all renters age 40-65 in 2008-2012. This represents a sample of individuals who are renting for 8-12 years of midlife. We focus on midlife prior to retirement age (when living conditions may be shifting and there is higher risk of mortality selection) and after young working ages (when income and tenure are more volatile). We fit the following Cox model:

|  | $h(t)=h_{0}(t)\times exp(\beta_{1}(R_{i}^{2000})\boldsymbol{+}{\boldsymbol{\beta}_{\boldsymbol{2}}\boldsymbol{(S}}_{\boldsymbol{i}}\boldsymbol{)+}{\boldsymbol{\beta}_{\boldsymbol{3}}\boldsymbol{(X}}_{\boldsymbol{i}}))$ | (1) |
| --- | --- | --- |

Where $h_{0}(t)$ is the baseline hazard function, $R_{i}^{2000}$ is continuous rent burden in 2000, $\boldsymbol{S}_{\boldsymbol{i}}$ is state of residence in 2000, and $\boldsymbol{X}_{\boldsymbol{i}}$ is a vector of individual, household, and neighborhood confounders measured in 2000: baseline age (continuous), educational attainment (less than high school, high school, some college, college or more), household income (continuous), U.S.-born (0/1), number of children (continuous), household size (continuous), marital status (0/1), living in the same place five years ago (0/1), veteran status (0/1), disability status (0/1), unemployed (0/1), number of bedrooms (continuous), residential building size (single-unit, mobile single-unit, 2, 3-4, 5-9, 10-19, 20-49, 50 or more units), tract-level median household income (continuous), and tract-level poverty rate (continuous). The coefficient $\beta_{1}$ in Equation 1 corresponds to the change in the hazard ratio for mortality (2000-2019) associated with a one percentage point increase in rent burden in 2000. To examine potential non-linear associations across the continuous domain of rent burden, we also use a penalized cubic spline. We additionally fit stratified models by race-ethnicity and gender.

Second, to estimate the association of within-individual rent burden changes (2000 to 2008-2012) with all-cause mortality (2008-2012 to 2019), we fit the following Cox model:

|  | $h(t)=h_{0}(t)\times exp(\beta_{1}(\Delta R_{i})\boldsymbol{+}{\boldsymbol{\beta}_{\boldsymbol{2}}\boldsymbol{(S}}_{\boldsymbol{i}}\boldsymbol{)+}{\boldsymbol{\beta}_{\boldsymbol{3}}\boldsymbol{(X}}_{\boldsymbol{i}}\boldsymbol{)+}\beta_{4}({R_{i}}^{2000})+\beta_{5}(\Delta I_{i}))$ | (2) |
| --- | --- | --- |

Where $h_{0}(t)$ is the baseline hazard function, $\Delta R_{i}$ is continuous change in rent burden, $\boldsymbol{S}_{\boldsymbol{i}}$ is state of residence in 2000, and $\boldsymbol{X}_{\boldsymbol{i}}$ is the same vector of confounders above. Here we use all renters aged 40-65 in the 2008-2012 ACS, so rent burden changes correspond to changes over the preceding 8-12 years. The coefficient $\beta_{1}$ in Equation 1 corresponds to the change in the hazard ratio for mortality (2008-2012 to 2019) associated with a one percentage point increase in rent burden in 2000. To examine potential non-linear associations across the continuous domain of changes in rent burden, we also use a penalized cubic spline. We additionally fit a model based on the subsample of renters who did not move between 2000 and 2008-2012.

We condition on two additional factors related to conceptualizing changes in rent burden as a life course exposure: initial rent burden (${R_{i}}^{2000}$) and household income in the 2008-2012 ACS relative to household income in the 2000 Census $(\Delta I_{i})$. By definition, rent burden depends on both rent and income. By conditioning on $\Delta I_{i}$ we aim to isolate mortality risk associated with burden fluctuations resulting from changes in *rent* ($\beta_{1}$) rather than changes in *income*. It is possible that the impact of rent burden changes on mortality may depend on initial rent burden (i.e., increasing from 30-40% burden may have a different impact on mortality than increasing from 40-50%) and income trajectories (i.e., increasing from 30-40% burden may have a different impact on mortality for individuals where household income has increased vs. decreased). We test for two-way and three-way interactions across these factors, but do not find statistically significant evidence of interactive effects. In sensitivity analyses, we also stratify this model by race-ethnicity, gender, income status in 2000 and 2008-2012 (consistently above vs. below the poverty threshold), smaller age windows (40-50, 50-65), and removing individuals who experienced large decreases in household income over the period (a decrease of more than 10% relative to household income in 2000). Results are substantively similar across all of these specifications, and we report estimates based on our pooled model above.

We weight observations in this model using inverse probability-of-censoring weights.(Cole and Hernan, 2008; Howe et al., 2016) We estimate these weights using a logistic regression model to predict selection into the sample in 2008-2012 as a function of baseline covariates in 2000. These weights correct for two sources of potential selection bias into our sample (assuming there are no unobserved predictors of these selection mechanisms not captured by our baseline covariates): mortality prior to 2008-2012 or transition to homeownership between 2000 and 2008-2012. Because relationships between baseline covariates and these two selection mechanisms are likely very different (e.g., high household income at baseline is likely associated with relatively high likelihood of transition to homeownership and relatively low risk of mortality), we test several flexible models for estimating these weights: logistic regression with penalized cubic splines for continuous predictors, gradient boosting machines (GBM), and non-parametric Bayesian additive regression trees (BART). We also test fitting models for each selection mechanism separately (mortality and homeownership) and summing the predicted weights. Results are substantively similar across all these tests (Figure S8).

Last, we examine whether this analysis is sensitive to treating all imputed values as true values, which may artificially deflate standard errors to the extent that missing values are imputed by internal Census edit/allocation procedures (Table S2). It is important to note that missing values for certain variables (e.g., household income) are not always imputed due to non-response but are often allocated using a logical set of deterministic rules: “some of the more common types of error included misread characters, misidentification of an income source, reporting subannual amounts such as monthly or weekly values, double reporting, or not reporting income at all.”(US Census Bureau, 2009) We conduct two tests for handling values with the edit/allocation flag in our analysis of rent burden changes across midlife because it is our smallest sample and also includes variables from the 2008-2012 ACS (Table S2). First, we compare point estimates from analyses using the entire sample with imputed values and the subset of complete-case respondents (i.e., no edit/allocation flags on any variables) (Figure S8, see above). Point estimates are very similar, suggesting that imputed values do not vary systematically across respondents in ways that might introduce bias to our analysis. Second, even if the edit/allocation procedure does not have substantive implications for our point estimates, treating the imputed values deterministically will deflate our standard errors to some degree. In Figure S8, our standard errors are only slightly larger in the complete-case analysis, suggesting that incorporating probabilistic multiply imputed values may not have substantive implications for our final analysis. We report results from a sensitivity analysis where we restrict our sample of rent burden changes to only respondents who do not have an edit/allocation flag for any key variable. We find that point estimates are virtually identical in this complete-case sample and standard errors only slightly larger than our primary sample where we treat all covariate values as true values (Figure S8).

*S2.2. Eviction and all-cause mortality*

To estimate the association of eviction with all-cause mortality, we use all renters over age 22 in 2000 (N=6.6 million) where the time-varying exposure is whether an individual was threatened with eviction (i.e., an eviction court filing without a judgment) (N=187,000), or evicted from their home (i.e., received an eviction judgment) (N=327,000). Using only links in the sample of PIK-matched evictions means that our estimates of the association between eviction and mortality will be conservative to the extent that we are treating some renters as unexposed (never evicted) when they should really be included in the exposed group (evicted) but are not because of incomplete matching. The time-varying outcome is all-cause mortality. There are three temporal issues inherent to this design: 1) eviction is a time-varying exposure (e.g., renters are exposed to filings/judgments at different times/ages), 2) the hazard ratio between those evicted and those not evicted may vary over time/age (e.g., the proportional hazards assumption of the conventional Cox survival model may not hold), and 3) the hazard ratio may vary based on time since eviction exposure (e.g., there is potentially a different acute/short-term vs. chronic/long-term effect of eviction on mortality risk).

The first issue results in a built-in selection bias if we were to simply compare cumulative mortality rates in those ever evicted vs. those never evicted from 2000-2016.(Hernan, 2010) In Kaplan-Meier curves of cumulative survival or a Cox model stratified by ever evicted vs. non-evicted, individuals in the *ever* *evicted group* have built-in “immortal time”—they cannot experience the outcome, all-cause mortality, prior to when they are evicted.(Suissa, 2008) In contrast, every individual classified in the *non-evicted group* is at risk of mortality at every age. This is often referred to as “immortal time bias” and necessitates a time-dependent design. The second issue may arise where experiencing an eviction at *age 60* may have a higher relative risk on mortality than experiencing an eviction at *age 55*. The third issue may arise where the relative risk of eviction on mortality varies between *one-year post-eviction* and *five-years post-eviction*, for example. These latter two issues might interact, such that the relative influence of an eviction on mortality five years later might depend on whether that eviction was experienced at age 55 or 60.

To account for these dynamics, we fit the following generalized linear model with the complementary log-log link to person-period data from 2000-2016 (Equation 3). The complementary log-log link preserves the proportional hazards property of the Cox model rather than the proportional odds property of the logit link. Results based on the logit link are virtually identical. Individuals ($i$) are indexed by age/year ($t$) as alive or dead by the end of the period. The exposure is whether the individual was threatened with eviction (i.e., an eviction court filing without a judgment) or evicted from their home (i.e., received an eviction judgment), or never threatened with eviction ($\boldsymbol{e}_{\boldsymbol{it}}$) at or before time $t$.

|  | $\mathrm{cloglog}\left( m_{it} \right)=\beta_{0}\boldsymbol{+}\boldsymbol{\beta}_{\boldsymbol{1}}\left( \boldsymbol{e}_{\boldsymbol{it}} \right)+\boldsymbol{\beta}_{\boldsymbol{2}}\boldsymbol{(}\boldsymbol{\delta}_{\boldsymbol{t}}\boldsymbol{)+}{\boldsymbol{\beta}_{\boldsymbol{3}}\boldsymbol{(S}}_{\boldsymbol{i}}\boldsymbol{)+}{\boldsymbol{\beta}_{\boldsymbol{4}}\boldsymbol{(X}}_{\boldsymbol{i}}\boldsymbol{)+}\varepsilon_{it}$ | (3) |
| --- | --- | --- |

In Equation 3, $\boldsymbol{\delta}_{\boldsymbol{t}}$ is a penalized cubic spline for continuous age/year since baseline (2000), $\boldsymbol{S}_{\boldsymbol{i}}$ is state of residence in 2000, and $\boldsymbol{X}_{\boldsymbol{i}}$ is the same vector of confounders above. We find no evidence that the association between eviction exposure ($\boldsymbol{e}_{\boldsymbol{it}}$) and mortality varies by age/year ($\boldsymbol{\delta}_{\boldsymbol{t}}$), so we use this simpler specification with no exposure-age interaction for our primary results.

*S2.3. Varying associations of eviction and all-cause mortality*

We use two general strategies to examine heterogeneity in the association between eviction events and mortality. First, we simply stratify our pooled model (Equation 3) by several factors. We stratify by cohort (ages 30-34, 50-54, and 70-74 in 2000), as background mortality rates are much lower at younger ages and might lead to different associations between eviction and mortality across the life course. We also stratify by race-ethnicity and gender, as evictions are heavily concentrated in non-white renting populations, especially those racialized as Black. Last, we tested two other factors where we did not find significant differences in stratified models. We interacted eviction events with a cubic spline for continuous time since exposure to test for differences in short-term vs. long-term associations with mortality, and we tested for differences in associations for those filed against or evicted multiple times, but confidence intervals are much wider for these estimates and not significantly different.

Our second strategy involves categorizing renters by predicted risk of ever being evicted ($\gamma_{i}$) based on logistic regression models stratified by race-ethnicity, gender, and baseline age (Equation 4). To allow for higher order interactions and non-linearities, we also predict eviction risk using gradient boosting machines (GBM) and non-parametric Bayesian additive regression trees (BART), additionally including baseline county of residence in 2000. Results are substantively similar.

|  | $\mathrm{logit}\left( \gamma_{i} \right)=\beta_{0}\boldsymbol{+}\boldsymbol{\beta}_{\boldsymbol{1}}\left( \boldsymbol{\delta}_{\boldsymbol{t}} \right)\boldsymbol{+}{\boldsymbol{\beta}_{\boldsymbol{2}}\boldsymbol{(X}}_{\boldsymbol{i}}\boldsymbol{)+}{\boldsymbol{\beta}_{\boldsymbol{3}}\boldsymbol{(S}}_{\boldsymbol{i}}\boldsymbol{)+}\varepsilon_{it}$ | (4) |
| --- | --- | --- |

We use these models to predict individual-level eviction propensities ($\gamma_{i}$) and stratify renters into three groups by propensity quartiles: Low Risk = bottom quartile, Moderate Risk = middle quartiles, and High Risk = top quartile. For simplicity in presenting results, we discretize renters into these three risk groups. We also examine risk deciles and use non-parametric smoothing methods to examine the relationship between cumulative mortality risk and continuous eviction propensity to ensure these discrete categories are not missing important variation.

To estimate whether the influence of eviction on mortality varies by risk of eviction, we fit similar models as in Equation 3 but stratified by these eviction risk groups. Given that there is still variation in continuous eviction risk within our discrete eviction risk groups, we also condition on the eviction propensity ($\hat{\gamma_{i}}$) predicted from Equation 4 in these stratified models. Under the ignorability assumption, conditioning on the propensity score ($\hat{\gamma_{i}}$) is as sufficient as conditioning on the full array of covariates ($\boldsymbol{X}_{\boldsymbol{i}}$) for the estimation of treatment effects.(Brand et al., 2019; Rosenbaum and Rubin, 1984)

|  | $\mathrm{cloglog}\left( m_{it} \right)={\beta_{0}\boldsymbol{+\beta}}_{\boldsymbol{1}}\left( \boldsymbol{e}_{\boldsymbol{it}} \right)+\boldsymbol{\beta}_{\boldsymbol{2}}\boldsymbol{(}\boldsymbol{\delta}_{\boldsymbol{t}}\boldsymbol{)}+\beta_{3}\boldsymbol{(}\hat{\gamma_{i}}\boldsymbol{)+}\varepsilon_{it}$ | (5) |
| --- | --- | --- |

The intuition behind these models is to examine possible heterogeneity in associations by “selection into eviction,” as the event of being evicted is often the culmination of many processes of cumulative disadvantage that also impact mortality risk. For example, we may find evidence of “negative selection,” where the impact of eviction on mortality risk is lower for those individuals at highest risk of eviction; these renters are likely already facing many challenges leading up to eviction that we do not observe, and which might shape mortality risk. In contrast, if the influence of eviction on mortality is constant across levels of eviction risk, then $\boldsymbol{\beta}_{\boldsymbol{1}}$ from Equation 5 will be similar across models stratified by eviction risk group. This approach of stratifying models by predicted exposure propensity has been used to estimate varying treatment effects of educational attainment on economic returns and parental divorce on educational attainment.(Brand et al., 2019; Brand and Xie, 2010)

*S2.4. Limitations and causal assumptions*

There are three primary limitations to our analysis related to data and measurement that we believe all lead to more conservative estimates. First, for all analyses of the association between eviction events and mortality, there is a possibility that estimates will be biased by systematic differences across the renter population in both the probability of being successfully assigned a PIK and the likelihood of living in a neighborhood where we observe close to 100% of eviction court records in our microdata comparing to official county-level aggregate reports. The Eviction Lab’s database of eviction records does not capture all formal evictions occurring between 2000-2016, and there may be a discrepancy between the total evictions reported by the state and the total reported by LexisNexis. Beyond gaps in the national coverage of the evictions database, many records could not be assigned a PIK, with state-level match rates varying from 78% (Washington, D.C.) to 48% (Hawaii). This is at least partly because the only personal identifiable information used in the matching process is name and address. This likely results in lower match rates among individuals who are extremely insecure in terms of housing stability; for example, an individual who moves very frequently may begin renting from a particular address and be evicted from that address before it is recorded in any source that might be used to match that individual to a PIK. This would result in a more conservative estimate of the association between eviction and mortality to the extent that such individuals would be included in our analysis as never evicted. To test for sensitivity to PIK match rates and eviction data coverage, we subset all analyses to only county-years where we have validated the evictions database of individual records against county- and state-reported aggregate case counts(Desmond et al., 2018b) and where PIK assignment rates exceed 75%. Results are nearly identical using this subset of validated county-years, and we use the entire matched sample across all county-years for all analyses. We do not find significant evidence that this potential bias may be impacting our results (Figure S9).

Second, there is the issue of measurement regarding time-varying confounders, which we only observe at baseline in the 2000 Census (e.g., household income). Ideally, we would measure time-varying confounders at every person-period, which we could then use to adjust for the time-varying risk of eviction in an inverse-probability weighting framework.(Hernán et al., 2001; Karim et al., 2014) To interpret our associations between eviction and mortality as causal estimates, this lack of information creates stronger assumptions related to no unmeasured confounding. Still, the 2000 long-form Census represents the most detailed set of renter data that has been linked to formal eviction records to date across the entire country, including many of the variables identified as the most important determinants of eviction risk in more localized studies.(Desmond and Gershenson, 2017)

Third, there is the issue of mortality measurement within the Numident file. With the inclusion of recent improvements to data collection and validation, Finlay and Genadek (2021) demonstrate how closely the Numident totals track with the CDC totals, especially during the COVID-19 pandemic. Still, it is possible that the Numident data miss some deaths, particularly in very marginalized groups (e.g., the unhoused population and anyone without an SSN). Indeed, a small fraction of SSN holders tracked in Numident do not have a death reported despite implied ages over 100 in 2021, which we assign as deaths at that age following Finlay and Genadek (2021). Again, this may downwardly bias our estimates of cumulative mortality for the evicted population. The Numident file also does not contain information on the cause of death, which inhibits a more nuanced examination of the mechanisms connecting eviction to premature mortality. Future research should explore linking evictions to cause-specific mortality via direct linkage to death certificates. Still, in terms of studying the impact of eviction on population health, all-cause mortality contains very low measurement error and much higher construct validity relative to common self-reported health outcomes collected in longitudinal surveys (e.g., self-rated health).

Across all analyses, the key assumption in interpreting these associations as estimates of causal effects (e.g., the average treatment effect of rent burden increases or eviction events on all-cause mortality) is ignorability: the assumption that the exposure is uncorrelated with unobserved factors that affect mortality risk. As described above, these associations are conditional on a large set of observed baseline confounders from the long-form 2000 Census; still, ignorability is a strong assumption especially when we do not observe time-varying confounders; for example, in considering a tenant evicted in 2010, we observe household income in 2000 but not every year between 2000 and 2010.

**Supplementary references**

Bond, B., Brown, J.D., Luque, A., O’Hara, A., 2014. The Nature of the Bias When Studying Only Linkable Person Records: Evidence from the American Community Survey. CARRA Working Paper Series 1, 1–30.

Brand, J.E., Moore, R., Song, X., Xie, Y., 2019. Parental divorce is not uniformly disruptive to children’s educational attainment. Proceedings of the National Academy of Sciences of the United States of America 116, 7266–7271. https://doi.org/10.1073/pnas.1813049116

Brand, J.E., Xie, Y., 2010. Who benefits most from college? Evidence for negative selection in heterogeneous economic returns to higher education. American Sociological Review 75, 273–302. https://doi.org/10.1177/0003122410363567

Cole, S.R., Hernan, M.A., 2008. Constructing Inverse Probability Weights for Marginal Structural Models. American Journal of Epidemiology 168, 656–664. https://doi.org/10.1093/aje/kwn164

Desmond, M., 2012. Eviction and the Reproduction of Urban Poverty. American Journal of Sociology 118, 88–133.

Desmond, M., Gershenson, C., 2017. Who gets evicted? Assessing individual, neighborhood, and network factors. Social Science Research 62, 362–377. https://doi.org/10.1016/j.ssresearch.2016.08.017

Desmond, M., Gromis, A., Edmonds, L., Hendrickson, J., Krywokulski, K., Leung, L., Porton, A., 2018a. Eviction Lab National Database: Version 1.0. Princeton University, Princeton, NJ.

Desmond, M., Gromis, A., Edmonds, L., Hendrickson, J., Krywokulski, K., Leung, L., Porton, A., 2018b. Eviction Lab Methodology Report: Version 1.0. Princeton University, Princeton, NJ.

Finlay, K., Genadek, K.R., 2021. Measuring All-Cause Mortality with the Census Numident File. American Journal of Public Health 111, S141–S148. https://doi.org/10.2105/AJPH.2021.306217

Hernan, M.A., 2010. The Hazards of Hazard Ratios. Epidemiology 21, 13–15. https://doi.org/10.1097/EDE.0b013e3181c1ea43.The

Hernán, M.A., Brumback, B., Robins, J.M., 2001. Marginal structural models to estimate the joint causal effect of nonrandomized treatments. Journal of the American Statistical Association 96, 440–448. https://doi.org/10.1198/016214501753168154

Howe, C.J., Cole, S.R., Lau, B., Napravnik, S., Eron, J.J., 2016. Selection Bias Due to Loss to Follow Up in Cohort Studies: Epidemiology 27, 91–97. https://doi.org/10.1097/EDE.0000000000000409

Karim, M.E., Gustafson, P., Petkau, J., Zhao, Y., Shirani, A., Kingwell, E., Evans, C., Van Der Kop, M., Oger, J., Tremlett, H., 2014. Marginal structural cox models for estimating the association between β-interferon exposure and disease progression in a multiple sclerosis cohort. American Journal of Epidemiology 180, 160–171. https://doi.org/10.1093/aje/kwu125

Rosenbaum, P.R., Rubin, D.B., 1984. Reducing bias in observational studies using subclassification on the propensity score. Journal of the American Statistical Association 79, 516–524. https://doi.org/10.1080/01621459.1984.10478078

Suissa, S., 2008. Immortal time bias in pharmacoepidemiology. American Journal of Epidemiology 167, 492–499. https://doi.org/10.1093/aje/kwm324

US Census Bureau, 2009. History: 2000 Census of Population and Housing (Volume 1). U.S. Government Printing Office, Washington, D.C.

Wagner, D., Layne, M., 2014. The Person Identification Validation System (PVS): Applying the Center for Administrative Records Research and Applications’ (CARRA) Record Linkage Software. CARRA Working Paper Series 1, 1–26.


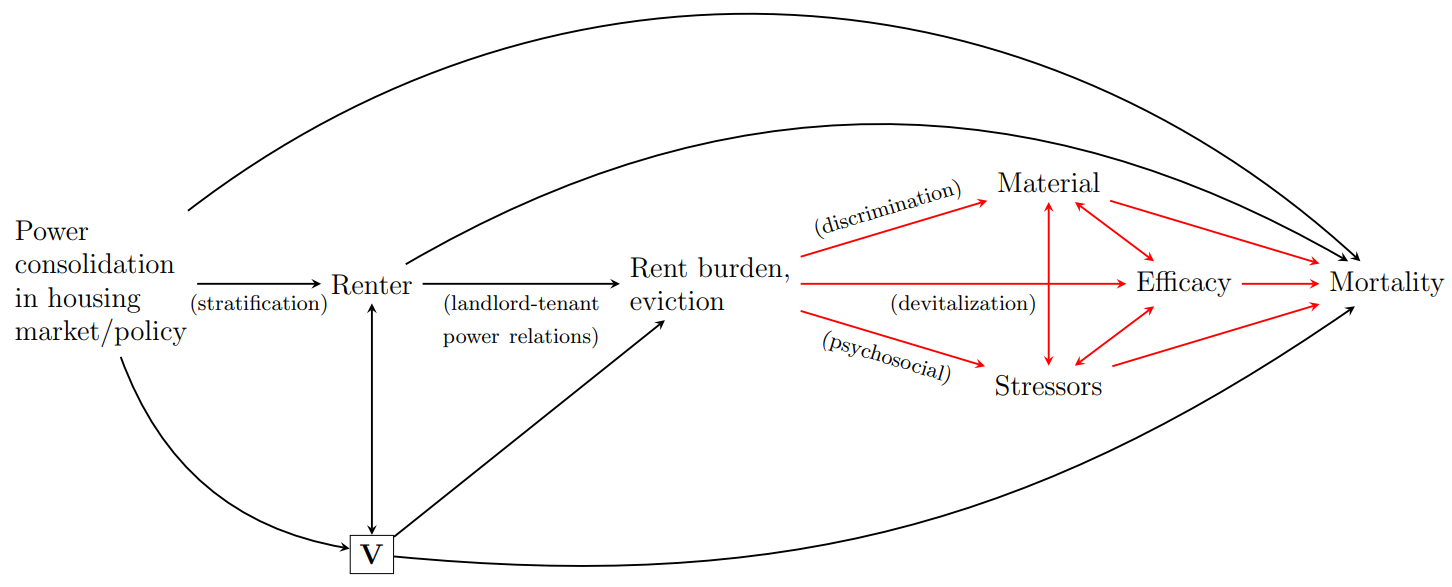
**Figure S1. Theoretical model.** Directed acyclic graph (DAG) situating rent burden and eviction as two mechanisms through which stratification in individuals owning vs. renting their housing is translated to differential mortality risk. The target estimand in our survival analyses is the conditional association of rent burden levels/changes and eviction on all-cause mortality among renters (i.e., the sum of the red arrows), where we condition on a vector of baseline renter, household, neighborhood, and state confounders (**V**) observed in the 2000 long-form Census. We discuss in the Limitations in Section S3 the conditions under which these associations may be interpreted as causal average treatment effects. “Material” refers to flexible material resources (e.g., money, approval for rental housing, access to credit, debt burden, food/healthcare insecurity), “Efficacy” refers to flexible social and political resources (e.g., perceived political capacity, social stigmatization, perception of self-worth, social networks), and “Stressors” refers to acute and chronic psychosocial harms that become embodied in physical and mental health outcomes.

**
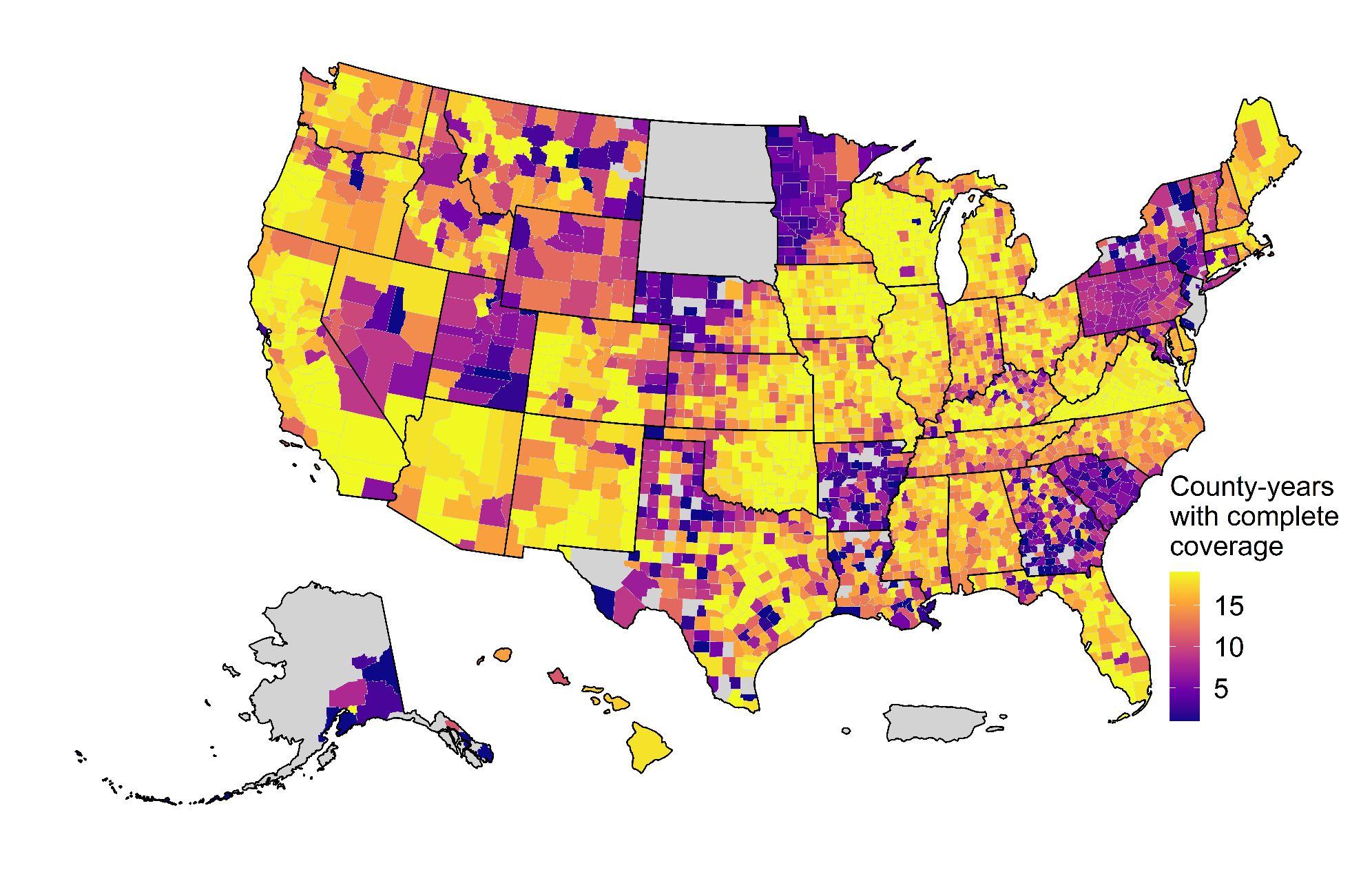
Figure S2. County-years with complete coverage of eviction court filings from 2000-2016.** Total county-years for which we are able to validate the total count of filings in the eviction filing microdata against aggregate filing statistics reported by county offices; see Section S2. Source: Desmond et al. (2018), available here: <https://data-downloads.evictionlab.org/#legacy-data/validated/>.

**
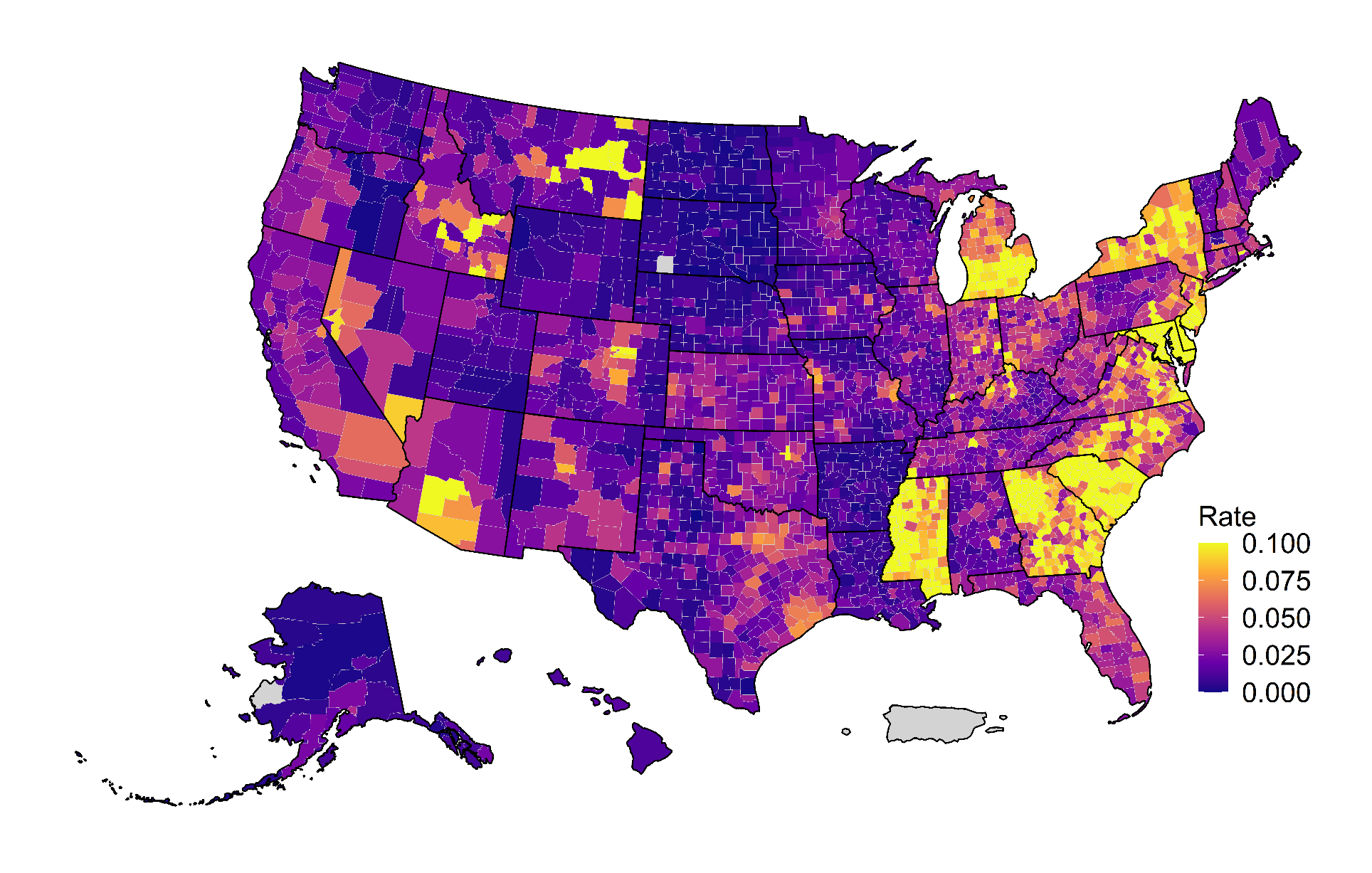
Figure S3. Geographic variation in the average annual eviction filing rate, 2000-2016.** The eviction filing rate is calculated as unique households filed against over unique renter households. Source: Gromis et al. (2022), available here: [https://data-downloads.evictionlab.org/#estimating-eviction-prevalance-across-us/](about:blank#estimating-eviction-prevalance-across-us/).


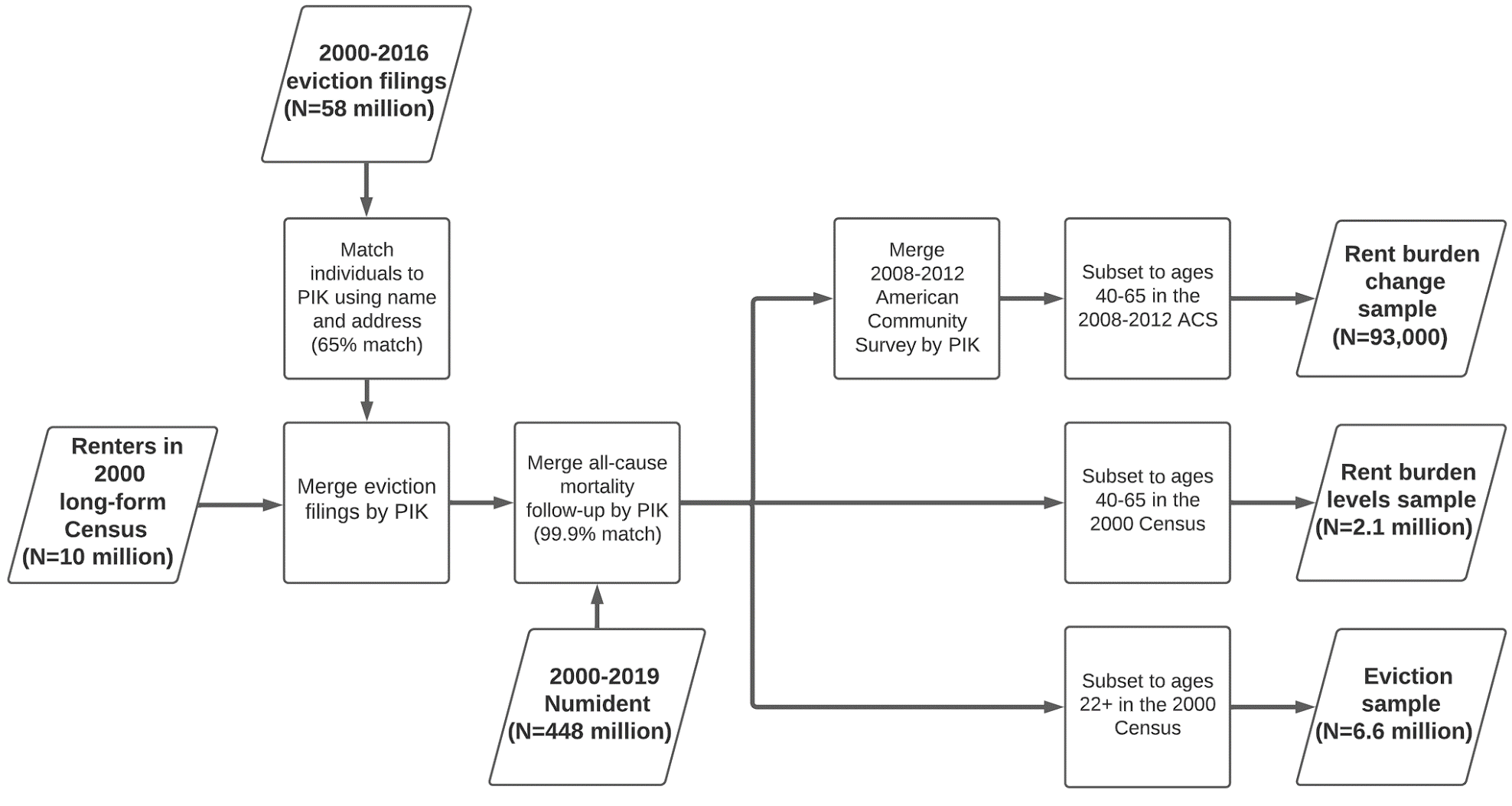
**Figure S4. Sample creation.** Flowchart of data processing beginning with the nationally representative population receiving the long-form 2000 Decennial Census (approximately 16% of the total population). Census Disclosure Review Board Approval Number: CBDRB-FY23-CES004-013. Sample sizes are rounded according to Census disclosure policy


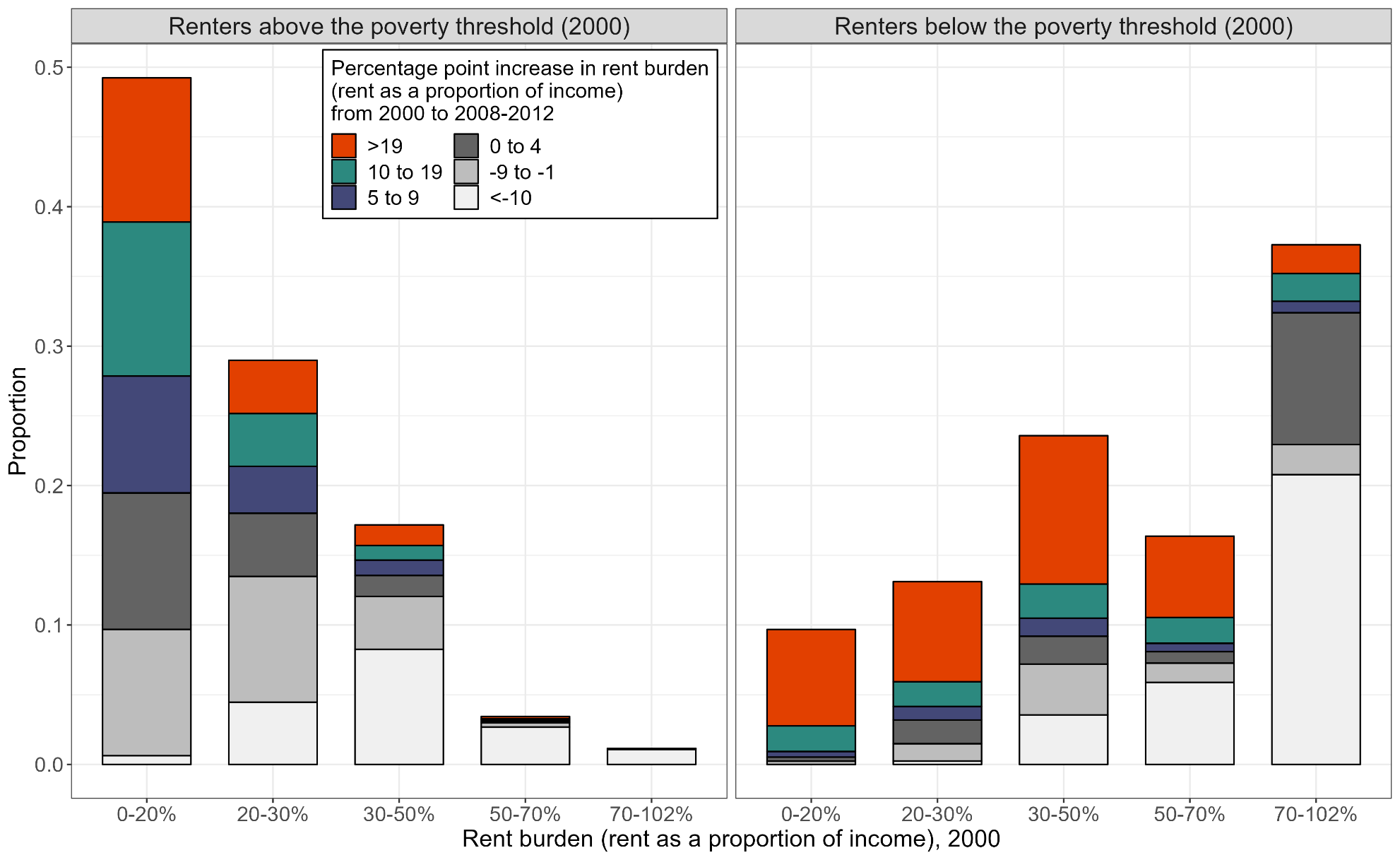
**Figure S5. Levels of rent burden in 2000 and changes in rent burden from 2000 to 2008-2012 for poor and non-poor persistent renters.** Colored bars indicate the proportions of each group that experienced different changes in rent burden by 2008-2012. Census Disclosure Review Board Approval Number: CBDRB-FY23-CES004-016. Sources: 2000 Census and 2008-2012 ACS.


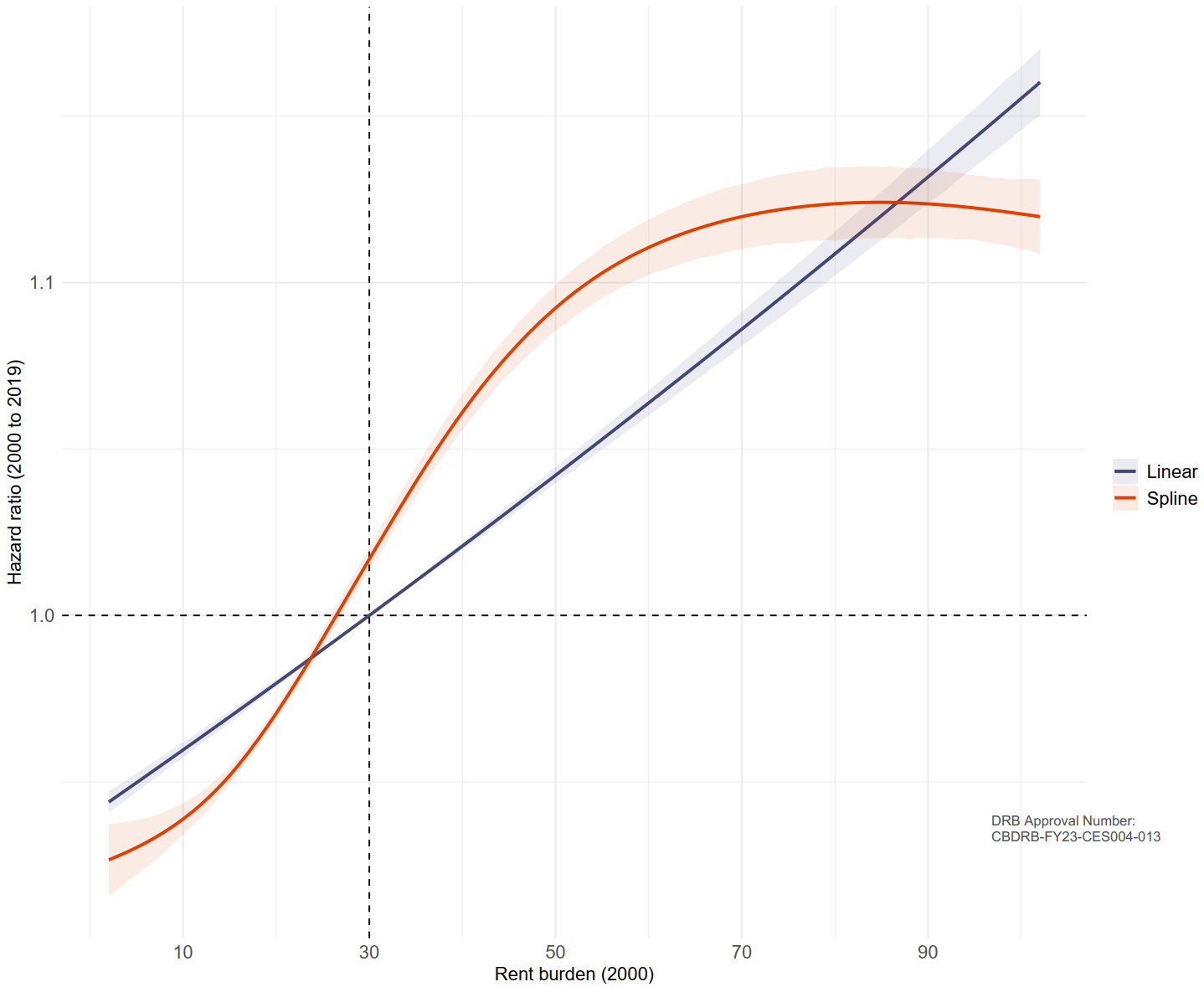


**Figure S6. Comparison of functional forms for levels of rent burden.** Estimates of the association between rent burden (2000) and all-cause mortality (2000-2019). Associations are reported based on a linear term and a penalized cubic spline for continuous rent burden (2000). Census Disclosure Review Board Approval Number: CBDRB-FY23-CES004-013. Sources: 2000 Census linked to 2021 Numident file.


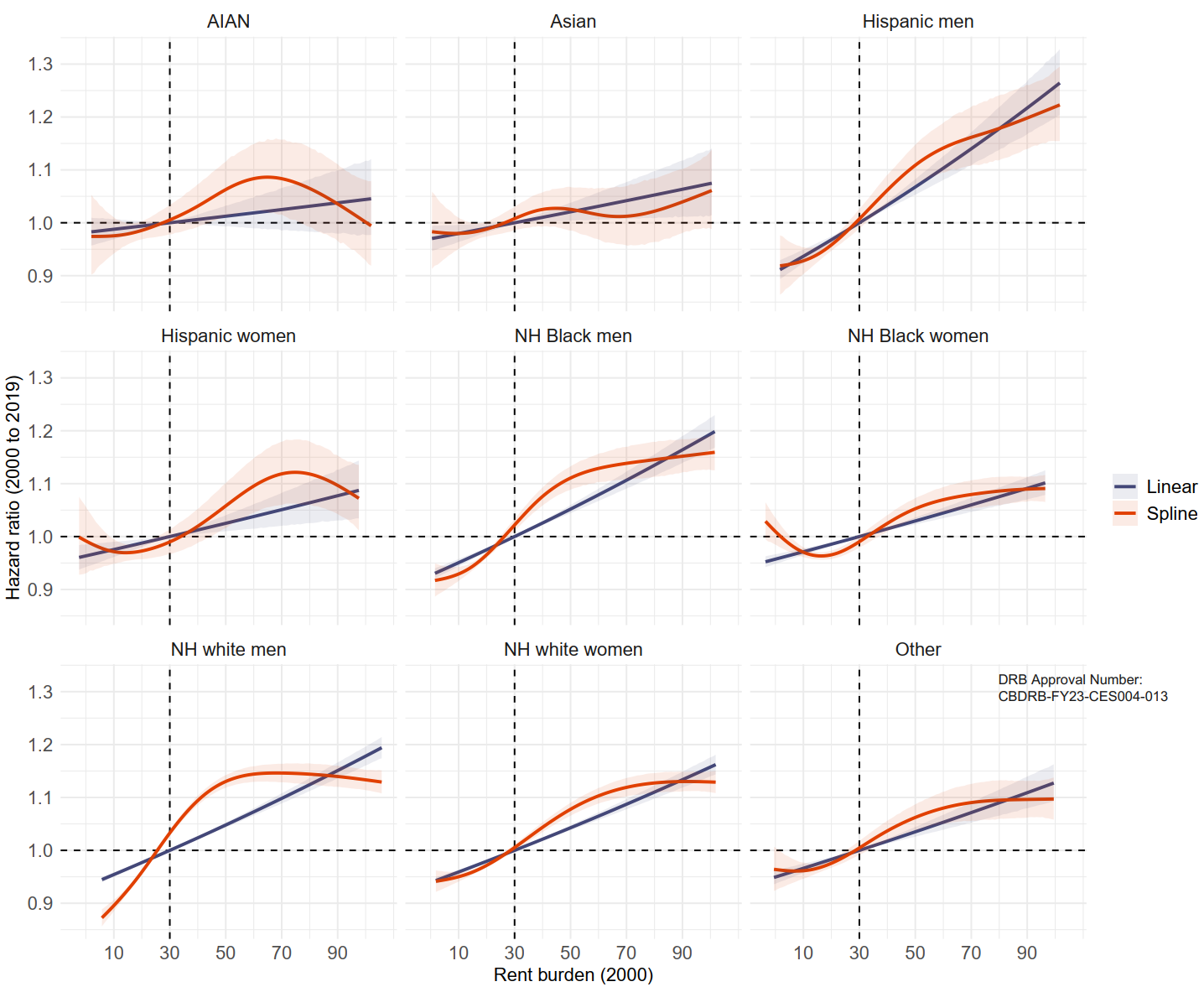
**Figure S7. Variation in the association between rent burden and mortality by race/ethnicity and gender.** Estimates of the association (hazard ratio) between rent burden (2000) and all-cause mortality (2000-2019) stratified by race-ethnicity-gender. Census Disclosure Review Board Approval Number: CBDRB-FY23-CES004-013. Sources: 2000 Census and 2008-2012 ACS linked to 2021 Numident file.


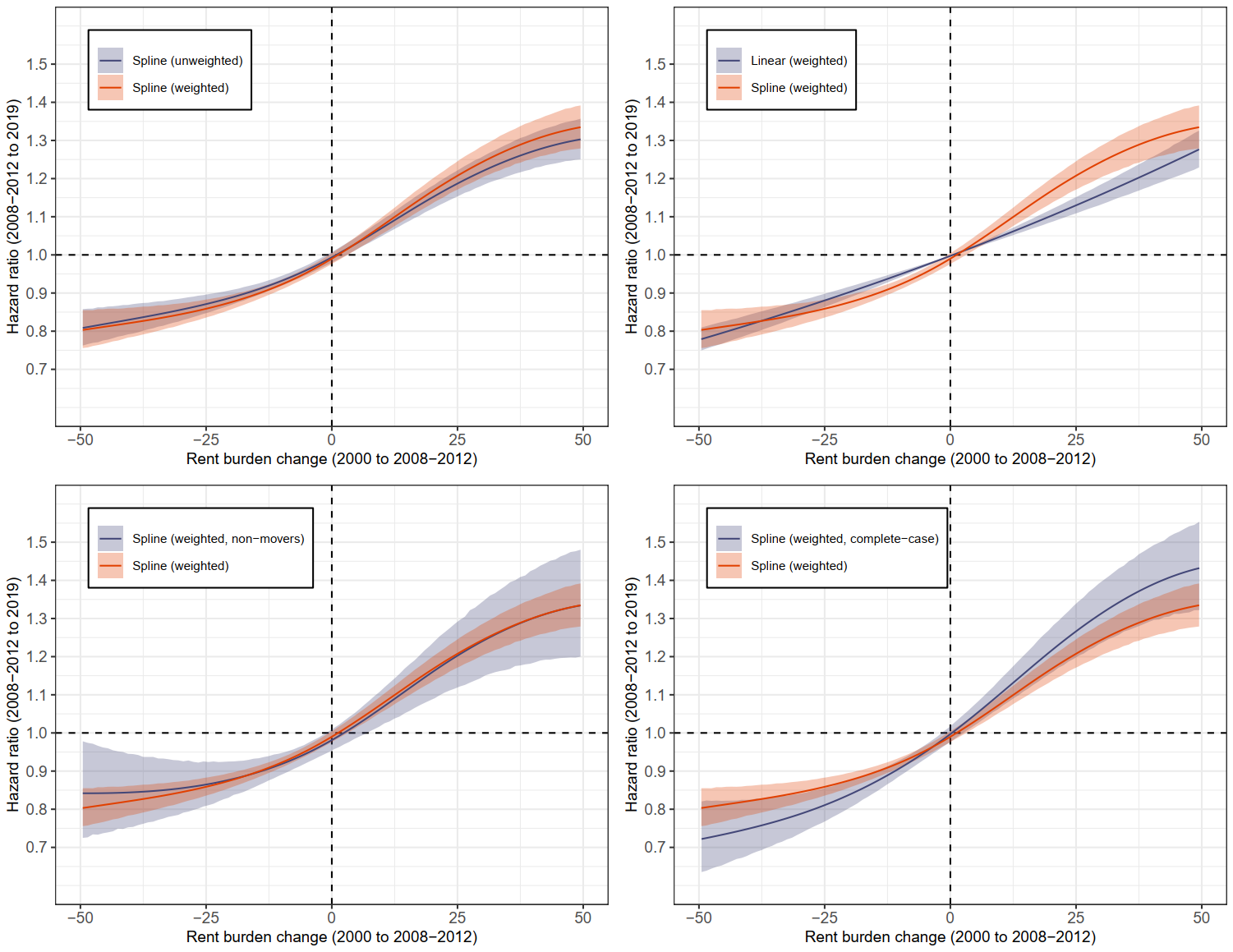


**Figure S8. Sensitivity analyses for estimating the association between rent burden change and mortality.** Estimates of the association between within-renter rent burden change (2000 to 2008-2012) and all-cause mortality (2008-2012 to 2019). Estimates are compared with/without inverse probability-of-censoring weights, with a linear vs. penalized cubic spline for continuous rent burden change, for a subset of renters who did not move between 2008-2012, and for a subset of complete-case renters who did not have an edit/allocation flag for any variables. Census Disclosure Review Board Approval Number: CBDRB-FY24-CES004-001. Sources: 2000 Census and 2008-2012 ACS linked to 2021 Numident file.

**
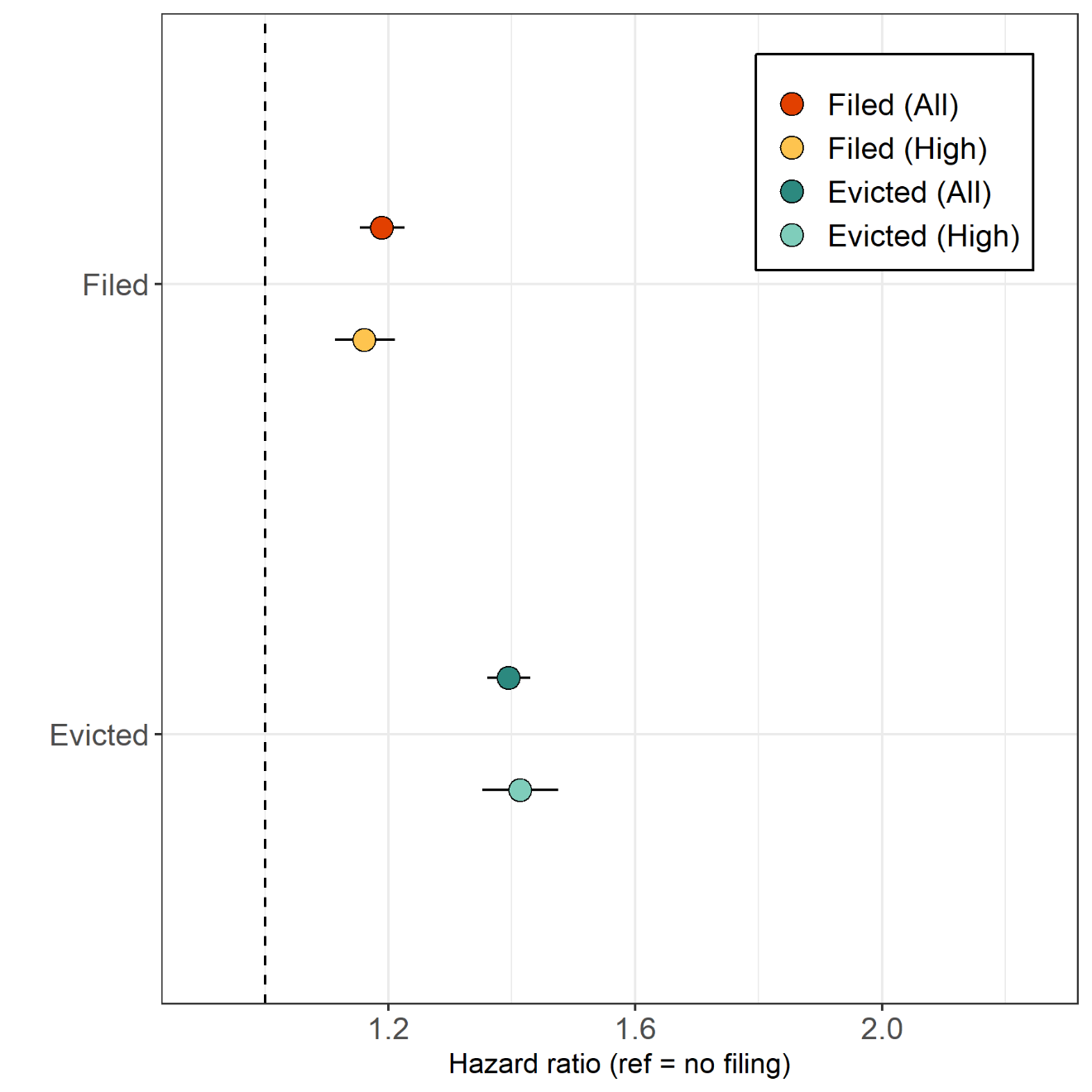
**

**Figure S9. Sensitivity analysis for varying rates of eviction data coverage and PIK assignment.** Estimates of the association between eviction events (eviction filing without a judgment, eviction judgment) and all-cause mortality (2000-2016), including estimates based on a subsample of renters initially living in Census tracts (2000) with high PIK match rates over the period (average across all years of over 75%) and high eviction data coverage (complete coverage in over >75% of years). Census Disclosure Review Board Approval Number: CBDRB-FY23-CES004-013. Sources: 2000 Census linked to 2021 Numident file and 2000-2016 eviction records.

|  | Hazard ratio (95% CI) |
| --- | --- |
| Baseline rent burden, 2000 |  |
| 10-point increase (linear) | 1.02 (1.02, 1.02) |
| Increase from 30% to 50% rent burden (spline) | 1.09 (1.09, 1.10) |
| Increase from 30% to 70% rent burden (spline) | 1.12 (1.11, 1.13) |
| Rent burden change, 2000 to 2008-2012 |  |
| 10-point increase (linear) | 1.05 (1.04, 1.05) |
| Increase of 0 compared to 10 points (spline) | 1.08 (1.05, 1.10) |
| Increase of 0 compared to 20 points (spline) | 1.16 (1.12, 1.19) |
| Filed against without a judgment |  |
| All renters | 1.19 (1.15, 1.23) |
| Ages 30-34 in 2000 | 1.20 (1.12, 1.27) |
| Ages 50-54 in 2000 | 1.14 (1.09, 1.19) |
| Ages 70-74 in 2000 | 1.22 (1.15, 1.29) |
| Non-Hispanic white men | 1.33 (1.25, 1.43) |
| Non-Hispanic white women | 1.32 (1.22, 1.42) |
| Non-Hispanic Black men | 1.08 (1.01, 1.16) |
| Non-Hispanic Black women | 1.08 (1.02, 1.15) |
| Hispanic men | 1.24 (1.03, 1.48) |
| Hispanic women | 1.44 (1.21, 1.69) |
| Asian | 1.74 (1.32, 2.24) |
| Other race/ethnicity/gender | 1.15 (1.03, 1.27) |
| 0-24^th^ eviction risk percentile | 1.59 (1.42, 1.78) |
| 25-74^th^ eviction risk percentile | 1.30 (1.24, 1.38) |
| 75-100^th^ eviction risk percentile | 1.10 (1.06, 1.15) |
| Evicted (filed against with a judgment) |  |
| All renters | 1.40 (1.36, 1.43) |
| Ages 30-34 in 2000 | 1.38 (1.32, 1.44) |
| Ages 50-54 in 2000 | 1.34 (1.29, 1.39) |
| Ages 70-74 in 2000 | 1.35 (1.27, 1.44) |
| Non-Hispanic white men | 1.52 (1.45, 1.59) |
| Non-Hispanic white women | 1.55 (1.47, 1.63) |
| Non-Hispanic Black men | 1.14 (1.06, 1.21) |
| Non-Hispanic Black women | 1.26 (1.20, 1.34) |
| Hispanic men | 1.25 (1.06, 1.48) |
| Hispanic women | 1.62 (1.35, 1.92) |
| Asian | 1.71 (1.31, 2.18) |
| Other race/ethnicity/gender | 1.35 (1.22, 1.48) |
| 0-24^th^ eviction risk percentile | 1.89 (1.66, 2.14) |
| 25-74^th^ eviction risk percentile | 1.51 (1.45, 1.58) |
| 75-100^th^ eviction risk percentile | 1.27 (1.23, 1.31) |

**Table S1. Associations of rent burden and eviction with mortality risk.** Data are hazard ratios estimated from Cox proportional-hazards models for rent exposures and discrete-time hazard models for eviction exposures. For eviction exposures, the reference group was renters never filed against. Adjusted for in each model: baseline age, race, ethnicity, gender, educational attainment, household income, nativity, number of children, household size, marital status, living in the same place five years ago, veteran status, disability status, being unemployed, number of bedrooms, residential building size, tract-level median household income, tract-level poverty rate, and state of residence. For exposure to rent burden changes, we additionally adjust for household income changes over the same period. Census Disclosure Review Board Approval Numbers: CBDRB-FY23-CES004-016, CBDRB-FY23-CES004-013. Sources: 2000 Census, 2008-2012 ACS, and 2000-2016 eviction records linked to 2021 Numident file.

|  |  | Sample | |
| --- | --- | --- | --- |
|  | Source | Adult renters in 2000 | Adult renters in 2000 linked to 2008-2012 ACS |
| Income | 2000 Census | 26.1 | 32.4 |
| Rent | 2000 Census | 13.3 | 11.6 |
| Education | 2000 Census | 5.5 | 3.7 |
| Employed | 2000 Census | 7.4 | 7.8 |
| Married | 2000 Census | 3.6 | 2.7 |
| Foreign-born | 2000 Census | 3.8 | 2.7 |
| Bedrooms | 2000 Census | 14.5 | 12.3 |
| Building size | 2000 Census | 4.4 | 3.4 |
| Same home for last five years | 2000 Census | 7.7 | 6.9 |
| Veteran | 2000 Census | 5.0 | 5.5 |
| Income | 2008-2012 ACS | --- | 28.4 |
| Rent | 2008-2012 ACS | --- | 20.0 |

**Table S2. Proportions (%) of respondents with edit/allocation flags for key variables.** Census Disclosure Review Board Approval Numbers: CBDRB-FY24-CES004-001. Sources: 2000 Census and 2008-2012 ACS.
